# Supplementary material for: Replication Protein A1 is essential for DNA damage repair during mammalian oogenesis
Source: bioRxiv. 2023 Jul 4:2023.07.04.547725. Preprint. [Version 1] doi: 10.1101/2023.07.04.547725 (PMC10349974; doi:10.1101/2023.07.04.547725)
Supplement: 1 [file NIHPP2023.07.04.547725V1-supplement-1.pdf]

bioRxiv preprint doi: https://doi.org/10.1101/2023.07.04.547725; this version posted July 4, 2023. The copyright holder for this preprint (which was not certified by peer review) is the author/funder, who has granted bioRxiv a license to display the preprint in perpetuity. It is made available under aCC-BY 4.0 International license.

Supplementary information

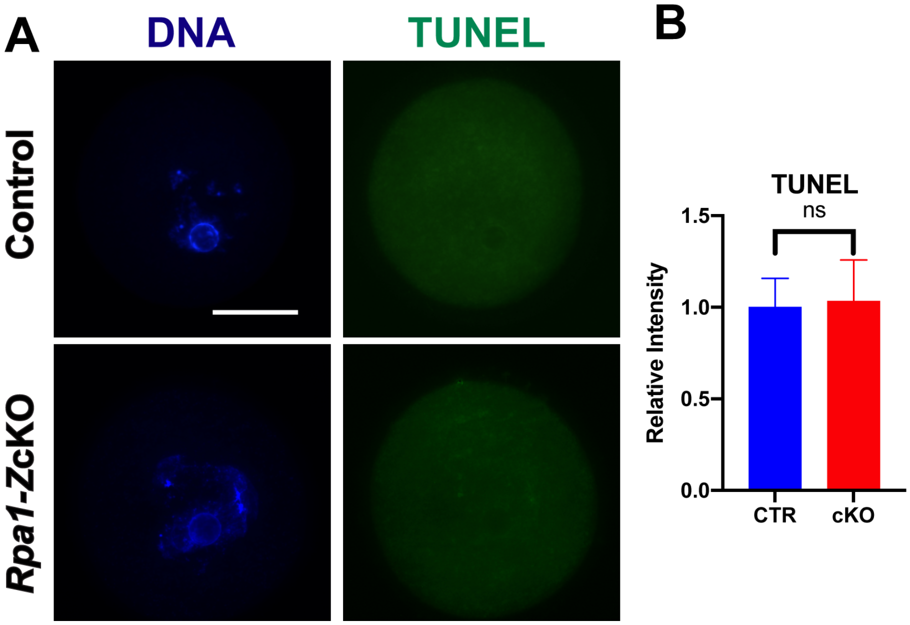

Fig. S1. No obvious DNA breaks were detected by TUNEL assay in *Rpa1*-ZcKO oocytes. (A) Representative images of TUNEL assay in control and *Rpa1*-OcKO GV oocytes. (B) Quantification of TUNEL signal in control (n=29) and *Rpa1*-OcKO (n=34) oocytes. Four mice per genotype, 4-5 weeks of age, were used in the experiment. Data are presented as mean ± SD. ns, not significant. Scale bar: 50 μm.

bioRxiv preprint doi: https://doi.org/10.1101/2023.07.04.547725; this version posted July 4, 2023. The copyright holder for this preprint (which was not certified by peer review) is the author/funder, who has granted bioRxiv a license to display the preprint in perpetuity. It is made available under aCC-BY 4.0 International license.

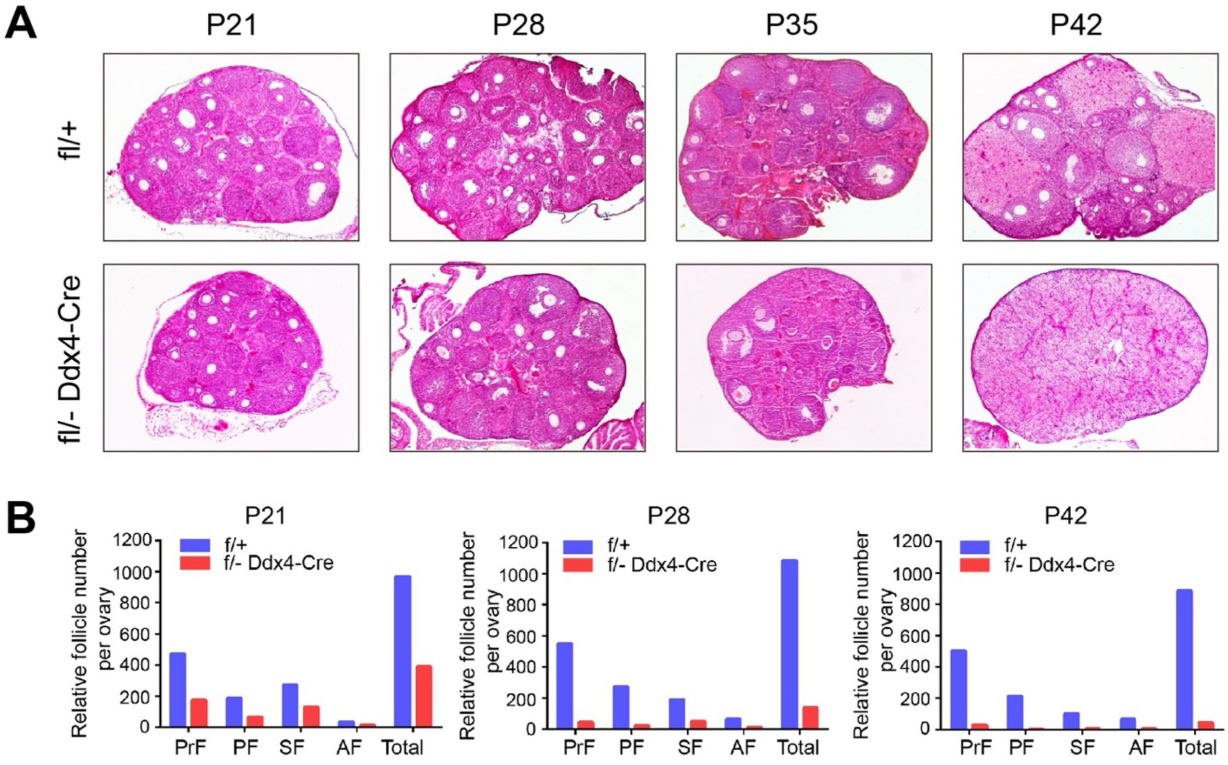

Fig. S2. Earlier and more severe loss of follicles, including primordial and primary follicles, were detected under the earlier driver *Ddx4-Cre*. (A) Histological analysis of ovaries at 3, 4, 5, and 6 weeks in control and OcKO females without hormone treatment. (B) Follicle quantification in control and OcKO females at different ages. PrF, primordial follicles; PF, primary follicles; SF, secondary follicle; AF, antral follicle. P, postnatal day. Four mice per genotype (1 per each age) were used in the experiment.

Table S1-*Rpa1* OcKO-RNAseq full list

Table S2-*Rpa1* OcKO-Significant-DEGs

Table S3-*Rpa1* OcKO-GO_analysis
